# Regulatory Mechanisms of miRNA Turnover: Insights into ZSWIM8-Mediated Target-Directed MicroRNA Degradation

**DOI:** 10.3390/biomedicines13092194

**Published:** 2025-09-08

**Authors:** Wenyao Zhang, Lixue Wang, Mohamed Yassine Demna, Jialong Xiong, Maoguo Luo, Yanfeng Wang, Feng Wang

**Affiliations:** 1 Key Laboratory of Molecular Medicine and Biotherapy, School of Life Science, Beijing Institute of Technology, Beijing 100081, China; zwy3200@163.com (W.Z.); demnayassine@bit.edu.cn (M.Y.D.); 3120221405@bit.edu.cn (J.X.); yf@bit.edu.cn (Y.W.); 2 College of Animal Sciences, Zhejiang University, Hangzhou 310058, China; wanglixue98@163.com; 3 Biological & Medical Engineering Core Facilities, School of Life Science, Beijing Institute of Technology, Beijing 100081, China; luomg@bit.edu.cn

**Keywords:** microRNA, AGO, TDMD, ZSWIM8, UPS

## Abstract

MicroRNAs (miRNAs), as an integral component of gene regulatory networks, play a critical role in post-transcriptional regulation, maintaining a dynamic balance between miRNA biogenesis and turnover essential for maintaining cellular homeostasis. The regulation of miRNA turnover, particularly through target-directed microRNA degradation (TDMD), is emerging as a key mechanism in gene expression control in response to physiological, developmental, and environmental changes. This process is mediated by the ubiquitin–proteasome system (UPS), where the E3 ligase ZSWIM8 functions as an adaptor to facilitate the recognition and degradation of Argonaute (AGO) proteins, essential components of the miRNA-induced silencing complex (miRISC), thus negatively regulating gene expression. The ZSWIM8–UPS axis contributes to the precise modulation of miRNA levels by targeting AGO proteins for degradation, thereby influencing miRNA stability and function. This review summarizes the mechanisms underlying ZSWIM8-mediated TDMD, its molecular interactions, and the potential therapeutic applications of targeting miRNA turnover pathways. By understanding the regulation of miRNA degradation, we aim to inform future strategies for the clinical manipulation of miRNA-based therapeutics.

## 1. Introduction

MicroRNAs are short non-coding RNA molecules that play important roles in regulating gene expression post-transcriptionally [1,2,3,4]. They bind to target mRNA sequences, leading to degradation or translational repression of the mRNA, thus controlling the synthesis of proteins essential for various cellular processes, such as development, differentiation, and immune responses. MicroRNAs are involved in various biological processes and have been found to target over 50% of the human genome [1]. The dysregulation of miRNA expression is associated with human diseases including cancer, neurodegenerative disorders, and cardiovascular diseases, positioning miRNAs as promising targets for gene therapy [5,6,7].

MicroRNAs bind to complementary regions within target mRNA sequence, leading to the degradation or translational repression of the target mRNA [8,9]. In mammals, miRNAs typically interact with the AGO protein complex, forming the miRISC [10]. Previous studies have shown that miRISC can directly degrade the target mRNA through the cleavage activity of the AGO protein [11,12].

The abundance of miRNAs is directly related to the functional activity of miRNAs, which is regulated by multiple mechanisms to act on transcriptional and post-transcriptional levels [13,14]. MicroRNAs are expressed differently in various cell types and during development, acting as meta-regulators of the gene to regulate activity at multiple cell levels, specifically transcription, translation, and protein degradation. Proteomic studies have uncovered the broad impact of a single miRNA on hundreds of targets [15], and likewise, a single gene can be regulated by multiple miRNAs. The actual elaborated insights on miRNAs have indicated that the aberrant expression level of miRNAs is connected with the initiation and development of human diseases, genetic disorders, and altered immune system function. MicroRNA processing defects can result in the occurrence and development of various diseases, such as tumorigenesis [16]. Therefore, miRNA expression profiles can be used as biomarkers for the onset and progression states of some diseases [17,18]. Interestingly, miRNA has emerged as a very promising drug target, and multiple microRNA drugs have been developed and used as gene therapy for genetic disorders [19,20].

Although insights into the regulatory function of miRNAs are beginning to emerge, the regulation of miRNA turnover has received less attention [21]. The stability of miRNAs is not only influenced by their biogenesis but is tightly regulated by mechanisms that control their degradation [22]. TDMD is a mechanism by which specific target RNAs induce miRNA degradation through recruitment of the UPS [23]. TDMD introduces a distinct regulatory layer that precisely adjusts miRNA levels in response to cellular needs and environmental signals [24,25]. The UPS is an ATP-dependent degradation system that recognizes and degrades ubiquitin-tagged protein substrates. The 26S proteasome, a multi-subunit protease, plays a key role in the degradation of polyubiquitinated proteins, thereby maintaining cellular proteostasis. In addition, the 26S proteasome can selectively remove misfolded proteins or non-essential proteins, which are closely related to many cell life processes, such as protein quality control, metabolism regulation, cell cycle control, and antigen presentation. In the UPS, the binding process of ubiquitin to the substrate is carried out by a multi-step cascade reaction consisting of E1, E2, and E3 enzymes. Dysfunction of the UPS can lead to a variety of diseases, such as neurodegenerative diseases, cardiovascular diseases, and cancer [26,27,28].

Cullin-RING E3 ubiquitin ligases (CRLs) are a large family of E3 ligases involved in the ubiquitylation of intracellular proteins. CRLs assemble different subunits, including Cullins, which act as molecular scaffolds and recruit specific target proteins. Zinc finger SWIM-type containing 8 (ZSWIM8) is a BC-box protein that acts as a substrate recognition module of CUL3, a Cullin protein. ZSWIM8 recruits AGO proteins and regulates miRNA turnover through TDMD. As a Cullin-RING E3 ubiquitin ligase adaptor protein, ZSWIM8 facilitates the ubiquitination and subsequent degradation of AGO proteins [29]. Additionally, ZSWIM8 is required for the targeted degradation of misfolded proteins, and its ortholog in *C. elegans*, EBAX-1, is involved in proper axon guidance.

Despite advances in understanding the function of miRNA, the regulatory mechanisms governing miRNA turnover, particularly through TDMD, remain underexplored. This review aims to provide a comprehensive overview of the ZSWIM8-mediated TDMD mechanism, its implications for gene expression regulation, and its potential therapeutic applications. By elucidating how miRNAs are selectively degraded through the UPS-mediated pathways, we can develop strategies for modulating miRNA levels in disease contexts, paving the way for novel therapeutic approaches targeting miRNA dysregulation.

## 2. MicroRNA Biogenesis, Function, and Regulation

**Figure 1:**
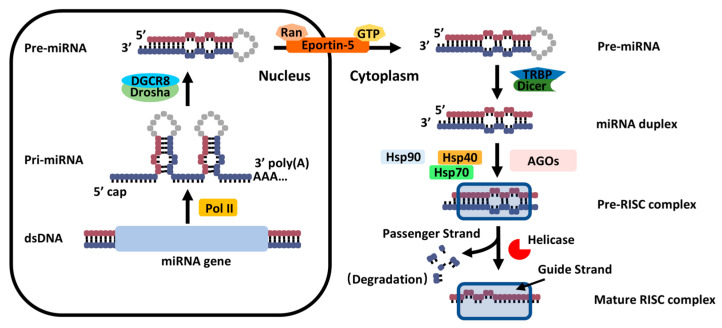
MicroRNA biogenesis pathway in animal. Canonical miRNA biogenesis begins with the generation of the pri-miRNA transcript. The microprocessor complex, comprising Drosha and DGCR8, cleaves the pri-miRNA to produce the pre-miRNA. The pre-miRNA is exported to the cytoplasm in an Exportin5/RanGTP-dependent manner and processed to produce an miRNA duplex. The mature miRNA duplex is loaded into RISC to form a pre-RISC complex. Finally, the pre-RISC complex is digested by helicase and forms a mature RISC complex.

**Figure 2:**
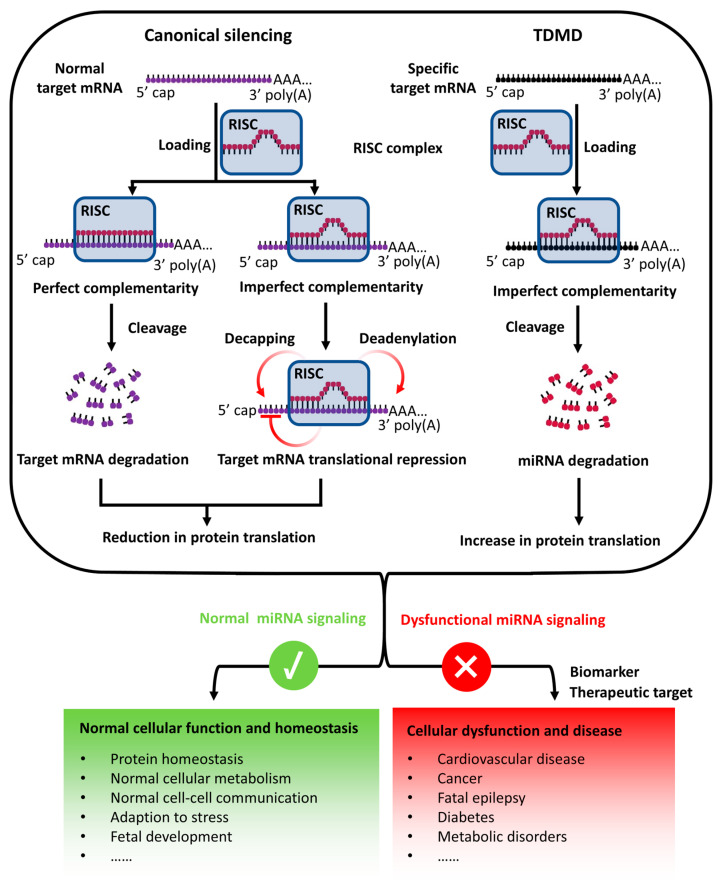
Role of miRNA signaling in cellular biology and disease. Normal target mRNAs are loaded into RISC and form either perfect or imperfect complementarity with miRNAs. Perfect complementarity leads to endonucleolytic cleavage of the target mRNAs, resulting in their degradation. Imperfect complementarity induces translational repression through multiple mechanisms, including deadenylation and decapping, ultimately leading to reduced protein production. By contrast, specific target RNAs, including artificial target RNAs, viral RNAs, and certain cellular RNAs, can trigger TDMD, which increases protein expression from those mRNAs. These mechanisms collectively maintain diverse cellular activities. Normal miRNA signaling ensures cellular homeostasis, including protein balance, metabolism, intercellular communication, and stress adaptation. By contrast, dysfunctional miRNA signaling contributes to cellular dysregulation and diseases, such as cardiovascular disorders, cancer, epilepsy, diabetes, and metabolic syndromes. Therefore, miRNA signaling represents both a diagnostic biomarker and a therapeutic target in multiple pathological contexts.

The biogenesis of classic miRNAs begins with their transcription as primary miRNAs (pri-miRNAs) by RNA polymerase II. These transcripts are processed in the nucleus by the Drosha–DGCR8 complex into precursor miRNAs (pre-miRNAs), which are subsequently exported to the cytoplasm via Exportin-5 for further maturation. In the cytoplasm, Dicer cleaves pre-miRNAs into mature miRNA duplexes, with one strand, the guide strand, incorporated into RISC (Figure 1) [21,30]. Within RISC, miRNAs bind to target mRNAs, primarily at their 3′ untranslated regions (UTRs), through base pairing. Depending on the degree of complementarity, miRNAs mediate either translational repression or mRNA degradation, thus regulating diverse cellular processes, such as development, differentiation, and immune responses (Figure 2) [8,31,32,33].

Despite their functional stability, miRNAs are subject to dynamic regulation to meet cellular demands and to maintain homeostasis. One such regulatory mechanism is TDMD, a process in which highly complementary target RNAs induce the degradation of miRNAs. Unlike canonical miRNA functions, TDMD involves the addition of A/U nucleotides at the 3′ end of the miRNA (tailing), a modification that destabilizes the miRNA and marks it for further processing [34,35,36]. This tailing is a crucial step in the selective degradation of miRNAs through TDMD, as it facilitates the exposure of the miRNA to exonucleases [37,38]. Following tailing, the miRNA undergoes trimming, a process by which the 3′ end of the miRNA is progressively shortened, further destabilizing the molecule and making it more susceptible to degradation [37]. This sequence of events is highly specific and is mediated by key proteins such as the ZSWIM8 ubiquitin ligase complex [29,39]. ZSWIM8 recognizes the RISC-bound miRNA with added A/U nucleotides, targeting it for ubiquitination and subsequent proteasomal degradation, thereby ensuring the removal of the miRNA and regulating its levels within the cell [21,29,39,40].

TDMD has been observed across diverse contexts, including interactions with viral and cellular RNAs. Evidence from model organisms highlights its evolutionary conservation: in *C. elegans*, the ZSWIM8 ortholog EBAX-1 mediates miRNA degradation, while in *Drosophila*, multiple endogenous transcripts direct specific miRNA decay that is critical for embryonic development [38,41,42]. In vertebrates, the long noncoding RNA Cyrano induces degradation of miR-7 in neurons to regulate neural development [43]. Moreover, viral RNAs exploit the TDMD pathway to modulate host miRNA abundance for their own benefit [44,45].

Understanding the balance between miRNA biogenesis and turnover, especially through pathways like TDMD, provides critical insights into the fine-tuning of gene regulation and its implications in health and disease. ZSWIM8-mediated TDMD represents a novel layer of miRNA regulation, highlighting its potential as a therapeutic target for diseases linked to aberrant miRNA activity [29,46,47].

## 3. The Balance of miRNAs Biogenesis and Turnover

The production and degradation of microRNAs (miRNAs) are tightly regulated processes essential for maintaining cellular homeostasis. MicroRNAs are among the fastest-produced and longest-lived cellular transcripts, with some miRNAs being released up to 10^5^ copies per cell under normal physiological conditions. Despite this high production rate, individual miRNAs exhibit considerable variability in their stability, ranging from minutes to weeks. This variability suggests the existence of a carefully orchestrated balance between miRNA biogenesis and turnover, which is crucial for their functional regulation in cells [35].

MicroRNA biogenesis begins with the RNA polymerase II transcription of miRNA genes, followed by processing steps that convert primary miRNA (pri-miRNA) into a mature, functional miRNA. This mature miRNA is incorporated into RISC, where it plays a pivotal role in regulating gene expression by silencing target mRNAs. However, for miRNAs to effectively fulfill this regulatory role, their abundance must be tightly controlled, both in terms of their production and their degradation. Thus, the balance between these two processes is essential for cellular function and organismal development [48,49].

The turnover of miRNAs is a dynamic and highly regulated process. It has been observed that the steady-state abundance of most miRNAs correlates well with their production rates, which indicates that the production rate is a major factor influencing miRNA levels. However, turnover also plays a critical role. During miRNA maturation, one strand of the precursor miRNA is incorporated into an AGO protein, where it is stabilized and protected from degradation. The other strand, known as the passenger strand, is usually degraded. The protective binding of AGO to miRNAs, particularly at the 5’ and 3’ unmodified ends, ensures their stability and facilitates their incorporation into the RISC complex [50,51,52].

However, the accumulation of miRNAs does not occur uniformly. In many cases, the accumulation of mature miRNAs occurs at a slower rate than the degradation of the passenger strand, with around 40% of miRNA duplexes being degraded even before AGO loading [36]. This suggests a highly regulated process wherein specific factors, such as AGO protein levels, influence the overall stability of miRNAs. For instance, the levels of AGO1 have been correlated with higher miRNA abundance, indicating that the formation of the RISC complex is critical for miRNA function and stability [53]. Interestingly, in species such as flies, miRNAs are sorted into distinct AGO complexes, each influencing the stability of the associated miRNAs differently. AGO2-bound miRNAs, for example, are significantly more stable than those bound to AGO1, likely reflecting the different functional roles these AGO proteins play [54,55].

The stability of miRNAs is also influenced by their susceptibility to degradation. Once bound to AGO, miRNAs can be exposed to nucleases if they are not properly protected or if the complex undergoes dissociation [56]. This is particularly relevant in the context of TDMD, a process that adds an additional layer of control over miRNA turnover [29,37,57]. Compelling evidence from experimental studies has demonstrated that TDMD actively reduces the abundance of specific miRNAs. For instance, Cyrano lncRNA induces degradation of miR-7 in mouse neurons, critically modulating neural development [43,58]. Similarly, viral RNAs, such as those from Herpesvirus saimiri, exploit TDMD to downregulate host miRNAs for their benefit [44]. More recently, ZSWIM8, the central E3 ligase complex in the TDMD pathway, was identified as indispensable for miRNA decay in mammalian and invertebrate systems, further confirming that TDMD is a conserved and functional mechanism of miRNA regulation [29]. TDMD is triggered when complementary target mRNAs interact with miRNAs in the RISC complex, leading to the degradation of the miRNA. Unlike canonical miRNA-mediated pathways where mRNAs are degraded upon miRNA binding, TDMD promotes the destabilization of the miRNA itself. This process is often initiated by 3′ non-templated nucleotide addition (tailing), such as oligo-adenylation or oligo-uridylation, which can subsequently serve as a substrate for trimming by exonucleases, including PARN (adenylated miRNAs) and DIS3L2 (uridylated miRNAs) [59]. Importantly, individual tailing events are not necessarily correlated with global changes in miRNA abundance [37,60,61].

TDMD is a relatively recent discovery and presents a novel mechanism for the regulation of miRNA levels. It is distinct from the classical model of miRNA-induced gene silencing, where miRNAs repress translation by binding to the 3’ UTRs of target mRNAs. In TDMD, the miRNA is cleaved and degraded, which prevents it from fulfilling its regulatory role. Importantly, this process is highly specific and requires complementary binding between the miRNA and its target mRNA, often involving structural rearrangements in the AGO complex [37,39,57,62].

While TDMD represents an important regulatory mechanism, it appears to operate independently of traditional miRNA–mRNA degradation pathways. TDMD and miRNA degradation through AGO2-mediated cleavage are not always co-dependent processes. In fact, evidence suggests that miRNA turnover and TDMD are two competing mechanisms, and the balance between these processes can be modulated by factors such as the relative abundance of the miRNA and its target mRNA [62,63]. This balance is particularly important in cellular contexts where miRNA levels must be rapidly adjusted in response to changing physiological conditions. For example, during cell differentiation or in response to environmental stress, cells may need to quickly alter miRNA levels to ensure proper gene expression.

Furthermore, while viral mRNAs can trigger TDMD by binding to host miRNAs, some cellular target RNAs also play an essential role in regulating miRNA levels. These cellular RNAs can induce miRNA degradation and thereby help maintain miRNA homeostasis within the cell. Studies have shown that mRNAs, such as Nrep in the mouse cerebellum, can directly interact with miRNAs like miR-29b, reducing their levels to maintain a physiological level of gene expression and to prevent excessive silencing [63]. These findings underscore the complexity of miRNA turnover and its regulation by cellular and external factors.

In summary, the balance of miRNA biogenesis and turnover is a highly intricate and dynamic process, regulated by a combination of miRNA production rates, AGO protein interactions, and turnover mechanisms such as TDMD. This balance ensures that miRNAs remain available for gene regulation while preventing the accumulation of aberrantly expressed or redundant miRNAs. The recent insights into TDMD, supported by strong experimental evidence across different species and contexts, have opened up new avenues for understanding how miRNAs are dynamically regulated, firmly establishing TDMD as a bona fide mechanism for controlling miRNA levels and adding an additional layer of complexity to the post-transcriptional control of gene expression [64,65,66]. Understanding these mechanisms will provide valuable insights into the functional roles of miRNAs in development, disease, and cellular stress response.

## 4. TDMD Mechanism Regulated by the UPS

In the intracellular environment, the degradation of microRNAs relies on intracellular RNA-degrading enzymes. However, most microRNAs can be protected by AGO proteins. The targeted degradation of AGO proteins plays a role in exposing microRNAs. In this regard, we summarize the evidence that the AGO protein interacts with several components of the UPS, including ZSWIM8, p97, and distinct subunits of the 26S proteasome. Understanding the functional impact of TDMD interactions with the UPS remains a significant challenge due to the limited knowledge of how the UPS recognizes ubiquitinated AGO and degrades it.

### 4.1. UPS

**Figure 3:**
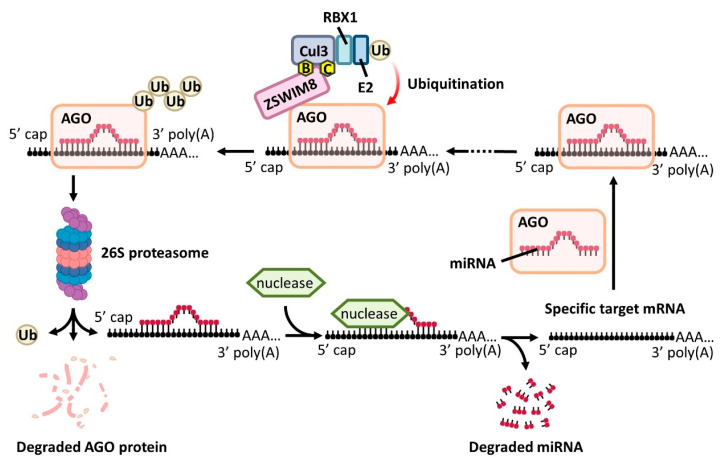
Simplified schematic representation of ZSWIM8-mediated TDMD. During TDMD, specific target mRNAs engage with AGO–miRNA complexes, which are subsequently recognized by the ZSWIM8 E3 ubiquitin ligase complex. ZSWIM8 promotes ubiquitylation of AGO proteins, marking them for proteasomal degradation. The disassembly of the AGO complex leads to miRNA release and subsequent nuclease-mediated decay, while the target mRNA is recycled for the next round of TDMD. Abbreviations: B, ELOB; C, ELOC; CUL3, Cullin 3; Ub, ubiquitin.

Protein degradation is accurately performed by the UPS [67,68]. Proteasomes are a large, multi-subunit complex found in both the nucleus and the cytoplasm [69]. Proteasomes in different tissues of higher animals exhibit distinct structures and are involved in different physiological processes. The specific and sophisticated degradation by 26S proteasome is determined by its structure, and the degradation center is called the 20S core particle with a double-stacked tubular structure complex formed by 28 proteins. The outer part of the seven-membered ring is composed of seven different α subunits in two layers, while the inner part is composed of seven different β subunits in two layers [67,70]. The inner layer of the 20S proteasome is composed of β-subunits, it has three peptidase activities, namely, caspase-like (β1, acidic amino acids), trypsin-like (β2, basic amino acids), and chymotrypsin-like (β5, hydrophobic amino acids), in order to directly degrade protein [69]. The UPS is a canonical pathway in the cell that targets proteins tagged with ubiquitin for proteolysis. Target proteins are covalently modified by ubiquitin (Ub) through an enzymatic cascade involving a Ub-activating enzyme (E1), a Ub-conjugating enzyme (E2), and a Ub-ligase enzyme (E3). Relevant studies have shown that Cullin-RING ligases (CRLs) account for the majority, with 95% of all E3 ligases. In mammals, there are at least eight highly conserved Cullin proteins, including CUL1, CUL2, CUL3, CUL4A, CUL4B, CUL5, CUL7, and CUL9. The Cullin proteins indirectly recruit substrates by associating with various interchangeable substrate receptors (SRs). The CUL3 family consists of three members: the RING finger protein RBX1, the CUL3 scaffold, and a Bric-a-brac/Tramtrack/Broad complex (BTB) protein. It has been discovered that the E3 ligase complex CUL3 ubiquitinates AGO, which is a key protein involved in TDMD. Recently, the protein ZSWIM8 has been identified as an adaptor that links AGO proteins to the ubiquitin ligase Cul3–Rbx1 complex, facilitating the ubiquitination and degradation of AGO proteins (Figure 3). The structure of human AGO2 suggests that extensive base pairing between miRNA and its target dislocates the 3’ end of miRNA from the PAZ domain. With increased base pairing, the central fissure of AGO2 becomes wider [71]. These two conformational changes, an empty PAZ domain and an enlarged central rift, provide a signature that ZSWIM8 can recognize as a signal for TDMD [29,39]. ZSWIM8 recruits the E2 ubiquitin-binding enzyme, which then polyubiquitinates the conserved lysine in AGOs. The addition of polyubiquitin signals the degradation of AGO by the proteasome. It is believed that the loss of its protective AGO partner would lead to rapid degradation of the now exposed miRNA [29,39].

### 4.2. AGO Proteins in TDMD

**Figure 4:**
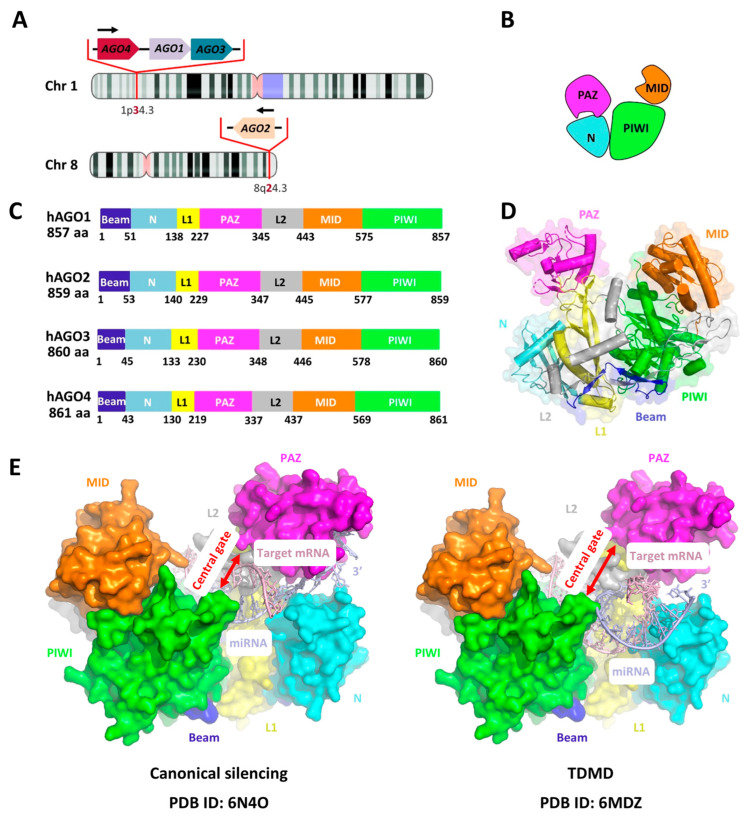
Genomic distribution, structural organization, and functional mechanisms of human AGO proteins. (**A**) Genomic information of human AGO1–4. (**B**) Schematic representation of conserved structural domains of human AGO proteins. (**C**) Domain organization and protein length of AGO1–4, highlighting similarities and differences among family members. (**D**) Structural model of human AGO2 (PDB ID: 4W5N). Protein structures were visualized using PyMOL 3.0. (**E**) Mechanistic comparison of AGO engagement with target RNAs in canonical silencing versus TDMD. In canonical silencing, seed and supplementary base-pairing stabilize the AGO–miRNA–mRNA complex with a closed central gate, leading to translational repression and/or mRNA decay. By contrast, extensive 3′-end pairing induces conformational changes in AGO, resulting in central gate opening, release of the miRNA 3′ end, and subsequent degradation through trimming, tailing, and ZSWIM8-mediated ubiquitin–proteasome pathways.

AGO proteins represent the core executors of RISC and are indispensable for miRNA-mediated gene regulation. In humans, four AGO paralogs (AGO1–4) are encoded on distinct chromosomal loci (Figure 4A), each sharing a conserved modular architecture, including the N, PAZ, MID, and PIWI domains (Figure 4B–D). Among them, AGO2 uniquely retains slicer activity. Structural analyses, such as the crystal structure of human AGO2 (PDB: 4W5N), have revealed a bilobal conformation, with the PAZ domain anchoring the 3′ end of miRNAs and the MID domain binding the 5′ phosphate [45]. This arrangement stabilizes miRNAs against exonucleolytic decay, allowing them to engage in canonical silencing.

In canonical silencing, AGO–miRNA complexes recognize targets primarily through seed (nt 2–8) and supplementary (nt 13–16) base-pairing, while the central cleft remains closed and the miRNA 3′ end securely docked in the PAZ pocket. This configuration facilitates translational repression and deadenylation of target mRNAs [8,45,71,72]. By contrast, TDMD emerges when miRNAs engage targets with extensive 3′-end pairing. Such interactions dislodge the miRNA 3′ end from the PAZ pocket and induce an “open” central cleft conformation of AGO (Figure 4E). These conformational changes destabilize the AGO–miRNA complex and mark AGO itself for ubiquitin-mediated turnover [29,39].

AGO degradation is tightly coupled to miRNA decay. Ubiquitination sites, such as K493 in AGO2 and homologous lysine in AGO1/3/4, have been identified as hotspots for UPS recognition [29,39]. Once AGO is ubiquitinated, the miRNA becomes unprotected and undergoes rapid tailing, trimming, and eventual clearance. Thus, AGO is not a passive carrier, but rather a molecular switch that determines whether an miRNA is stabilized or degraded.

The dual role of AGOs—guardians of miRNA stability in canonical silencing and substrates of proteasomal degradation during TDMD—positions them at the heart of small RNA biology. Moreover, the interplay between AGO structure, RNA pairing patterns, and post-translational modifications ensures that miRNA fate is tightly regulated in response to cellular signals. Understanding how AGOs transition between protective and degradative states is therefore crucial for dissecting the mechanistic integration of RNA silencing with proteostasis.

### 4.3. ZSWIM8

**Figure 5:**
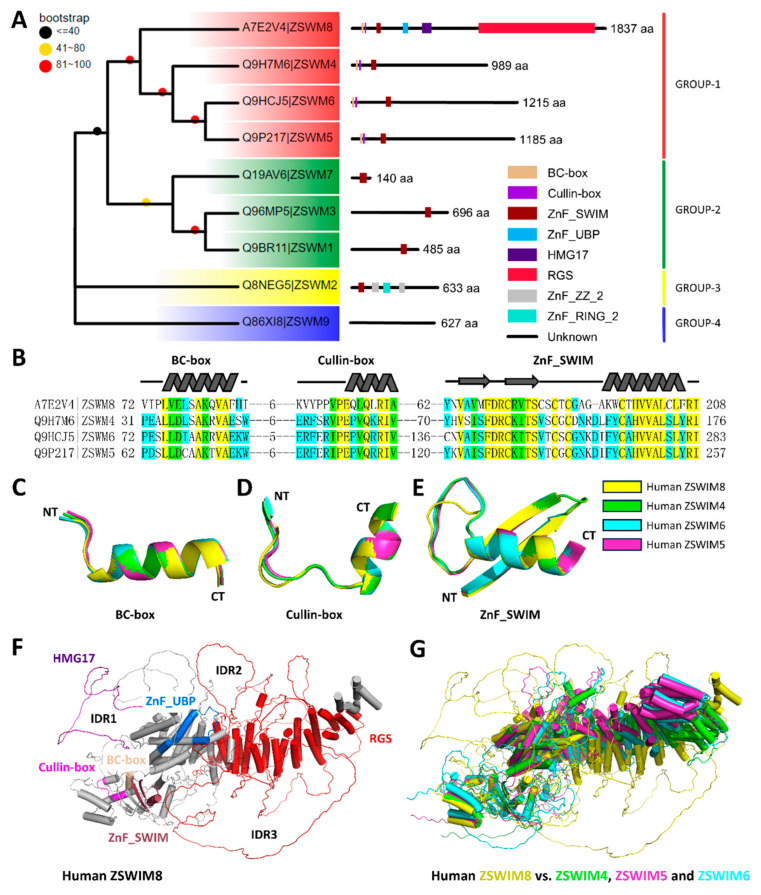
Structural analysis of human ZSWIM family. (**A**) Homologous evolutionary tree and domain organization of human ZSWIM family. (**B**) Secondary structure and conservative analysis of the BC-box, the Cullin-box and the ZnF_SWIM domain in ZSWIM8, ZSWIM4, ZSWIM5, and ZSWIM6. (**C**–**E**) The comparison of the BC-box, the Cullin-box and the ZnF_SWIM domain structures were predicted by Alphafold2 and rendered by PyMOL. (**F**) The structure of human ZSWIM8. (**G**) The comparison of human ZSWIM8, ZSWIM4, ZSWIM5, and ZSWIM6 structures were predicted by Alphafold2 and rendered by PyMOL.

Zinc finger proteins (ZFPs) are a class of proteins characterized by a small protein sequence that coordinates one or more zinc ions, forming a distinct zinc finger structure [73,74]. Initially identified in studies of *Xenopus laevis*, ZFPs are ubiquitous across different cell types and play pivotal roles in various biological processes. Their functionality stems from the zinc finger motif, which enables binding to DNA, RNA, and proteins, thus facilitating a wide range of cellular functions [75]. Human Zinc finger SWIM-type containing 8 (ZSWIM8) is a recently identified member of the ZSWIM family, distinguished by the presence of a novel zinc finger domain known as ZnF_SWIM. The full-length ZSWIM8 protein comprises 1837 amino acids (Figure 5). The ZSWIM family includes nine proteins (ZSWIM1–ZSWIM9), among which ZSWIM4, ZSWIM6, and ZSWIM5 exhibit relatively higher sequence similarity to ZSWIM8 (Figure 5A) [76]. Notably, these proteins share three conserved structural domains: the BC-box, the Cullin-box, and the ZnF_SWIM domain (Figure 5B–E). The SWIM domain of ZSWIM8 is characterized by a zinc-coordinating motif, oCxCxNCxH [77,78], which is integral to its structural and functional properties. This specific zinc chelation is crucial for the protein’s ability to mediate its interactions and to contribute to cellular regulation processes. It has been found to be expressed in the frontal cortex and 21 other tissues. The BC-box, the Cullin-box, and the ZnF_SWIM domains are in close proximity to each other, forming an interface that suggests a cooperative functional interaction (Figure 5F). This phenomenon has also been observed in ZSWIM4, ZSWIM6, and ZSWIM5 (Figure 5G). Importantly, human ZSWIM8 was previously reported to function as a substrate adaptor of a CRL containing Elongin B (ELOB) and Elongin C (ELOC) [79]. The homolog of ZSWIM8 is *C. elegans* Elongin BC-binding axon regulator-1 (EBAX-1), which belongs to an uncharacterized BC-box protein family conserved from invertebrates to humans [79]. EBAX-1, as a substrate-recognition subunit in the Elongin BC-containing Cullin-RING ubiquitin ligase (CRL), is involved in the targeted degradation of some unstable or misfolded proteins to destabilize miRNAs [80]. For instance, in neurons of *C. elegans*, EBAX-1, in cooperation with DAF-21, can regulate and control the protein quality of the SAX-3/Robo receptor to resist the occurrence of neurological diseases [79]. For instance, in the neuron of *C. elegans*, EBAX-1 cooperates with DAF-21 to regulate the protein quality of the SAX-3/Robo receptor, thereby protecting against neurological defects [79]. Consistently, human ZSWIM8 exhibits a similar substrate preference toward a Robo3 mutant receptor implicated in horizontal gaze palsy with progressive scoliosis (HGPPS) [79]. Structurally, EBAX-1/ZSWIM8 contains two N-terminal motifs—the BC-box and the Cullin-box—followed by a SWIM (SWI2/SNF2 transcription factor and MuDR transposase) domain and several conserved regions without clear homology to known domains [77,81].

ELOB and ELOC, two distinct proteins with sizes ranging from 10 kDa to 20 kDa, are components of the BC-box type Cullin-RING E3 ligase (CRL) associated with CUL2 and CUL5 [82]. CUL2/5 assembles ELOB, ELOC, the RING-Box protein Rbx1/Rbx2, and the BC-box protein as scaffold proteins. CUL2 and related Rbx1 proteins can form a heterodimer; similarly, CUL5 can also form a heterodimer with other related proteins. During the reconstitution of ubiquitin ligases, the BC-box protein can directly recruit the heterodimer to form a complex [83]. The ELOB and ELOC complex acts as an adaptor, linking a BC-box protein recognition subunit to the RING finger domain of the heterodimers to recruit and activate an E2 for specific substrate ubiquitination [81]. The interaction of the BC-box protein with the heterodimers is governed by specific regions, known as CUL2- or CUL5-boxes, located immediately downstream of their BC-boxes [83].

Human ZSWIM8 is a key BC-box protein that regulates the mechanism of miRNA turnover [39,84]. It is also associated with ELOB and ELOC and functions as a substrate adaptor of a Cullin-RING E3 ubiquitin ligase (CRL) [79,81]. Studies have shown that human ZSWIM8 has no functional response when CUL2 and CUL5 are inactivated [39]. By contrast, inactivation of CUL3 impairs the turnover of ZSWIM8-related miRNAs, resulting in the accumulation of miR-7. Accordingly, these results suggest that CUL3 may be a constituent of the ZSWIM8–ELOB–ELOC CRL complex, rather than CUL2 or CUL5 [39].

Human ZSWIM8 and its homologs form a conserved family of substrate-recognition subunits of CRLs that maintain normal homeostasis in different types of cells. In *Drosophila*, the ZSWIM8 homolog (CG34401 or Dora) regulates R7 photoreceptor axons and oogenesis [85,86]. Mouse and human ZSWIM8 are also widely expressed in the brain [87]. Additionally, mouse ZSWIM8 promotes the degradation of a human Robo3(I66L) mutant protein associated with HGPPS. Furthermore, the human homolog ZSWIM8 has been reported to interact with Ataxin 1 and Atrophin 1, two spinocerebellar ataxia-causing proteins [88]. Exploring the role of ZSWIM8 family members in the vertebrate nervous system, both during development and disease, would provide valuable insights.

### 4.4. Interaction Between the UPS and TDMD

The UPS has emerged as a central regulator of TDMD by controlling the stability and turnover of AGO proteins. Since AGO proteins act as the protective carriers of miRNAs, their selective degradation provides a key mechanism by which TDMD promotes miRNA clearance (Table 1).

Early studies sought to define the protein partners of AGO that could explain its regulation by the UPS. Using spectroscopic and genetic approaches, Shi et al. [39] and others [29,39,79] have identified ZSWIM8, a conserved BC-box protein, as a substrate adaptor for a CUL–RING ligase (CRL) complex containing ELOB and ELOC (Figure 4B). Through its N-terminal regions, ZSWIM8 recognizes AGO proteins and mediates their ubiquitination. This recognition appears to be independent of miRNA 3′-end modifications, since tailing and trimming of miR-7 and other miRNAs were shown to have little impact on ZSWIM8 recognition [29,89,90]. These findings have shifted the focus of TDMD research from miRNA modifications to the post-translational regulation of AGO proteins themselves.

Functional studies have further underscored the role of the UPS in TDMD. Inhibition of the ubiquitin-activating enzyme E1 with TAK-243, or proteasome inhibition with MG132, has led to the accumulation of miR-7 in HEK293 cells [29,90]. This result indicates that both ubiquitination and proteasomal degradation are indispensable for miRNA clearance during TDMD. Since miRNA degradation requires the destabilization of AGO proteins, these results support a model in which the UPS-mediated turnover of AGO drives TDMD.

Molecular evidence has revealed that ubiquitin conjugation occurs on the specific lysine of AGO proteins. Structural studies have identified 25 surface-exposed lysines in AGO2 conserved across multiple AGO homologs [91,92,93,94]. Among them, K493 was shown to be critical: mutation of this site markedly impaired miR-7 degradation [29,39]. Homologous residues in AGO1 (K491), AGO3 (K494), and AGO4 (K485) were also detected as ubiquitination sites in large-scale proteomic surveys, suggesting a conserved ubiquitination-dependent regulatory mechanism across the AGO family.

AGO stability is also influenced by molecular chaperones that functionally intersect with the UPS pathway. HSP90, an essential co-chaperone for RISC assembly, facilitates small RNA loading and protects AGO1/AGO2 from proteasome-mediated degradation [95]. Inhibition of HSP90 with geldanamycin resulted in reduced AGO levels, an effect reversed by MG132 treatment. These results suggest that AGO protein levels reflect a balance between chaperone-mediated stabilization and UPS-driven degradation.

Genetic and transcriptomic studies have further supported the role of the UPS in AGO regulation. Overexpression of miRNAs in *Drosophila* or mammalian cells induces accumulation of AGO1, resembling phenotypes caused by disruption of the UPS pathway [96]. Knockdown of the UPS components, including E2 (Uev1A), 19S subunits (Rpn9, Rpn11, Rpn8), and 20S subunits (α7, α1, α6, α4), as well as direct proteasome inhibition, all lead to AGO2 accumulation [97]. Together, these findings reinforce the requirement of the UPS for AGO protein turnover and highlight the broad involvement of multiple proteasomal subunits in this process.

Finally, ZSWIM8 itself shows extensive connections with proteasome subunits. Genetic screens have revealed its interaction with PSMA8 (Rpn12) and PSMC1, both components of the 19S regulatory particle [29,39]. Subsequent studies have extended these findings to other subunits, such as PSMA1, PSMD7, and PSMD11. Co-expression analyses have further confirmed correlations between ZSWIM8 and PSMC2, PSMC1, PSMD14, PSMD7, and PSMC5. These data suggest that ZSWIM8 collaborates closely with the 26S proteasome machinery to promote AGO ubiquitination and degradation. However, the precise molecular interfaces through which ZSWIM8 engages the proteasome remain to be determined.

In summary, the UPS regulates TDMD through a coordinated mechanism: ZSWIM8 recognizes AGO proteins and directs their ubiquitination on specific lysines, leading to their degradation by the 26S proteasome. This process destabilizes AGO–miRNA complexes, thereby facilitating selective miRNA decay. While considerable evidence supports this model, future studies are required to define the structural basis of ZSWIM8–proteasome interactions and to determine whether additional adaptor proteins contribute to AGO turnover during TDMD.

**Table 1:** Regulation of miRNA Decay and AGO Protein Stability: Mechanisms and Experimental Evidence.

miRNA	Target	Organism	Experimental Condition	Effect on TDMD	Reference
miR-7	AGO2	Human	Inhibitor (TAK-24, MG132)	Accumulation of miR-7	[29,39,79]
miR-7	AGO2	Human	K493 mutation	Loss of miR-7 degradation	[29,39]
miRNAs	AGO proteins	Human	HSP90 inhibitor (geldanamycin)	HSP90 protects AGO1/AGO2 from UPS degradation	[95]
miRNAs	AGO1	Drosophila	UPS component knockdown/inhibition	AGO accumulation; miRNA turnover impaired	[96,97]
—		Human	Genetic screen; co-expression	ZSWIM8 interacts with proteasome	[29,39]

## 5. Potential Drug Targets for TDMD in the UPS

The UPS and TDMD pathway both play crucial roles in cellular function and have been implicated in various diseases, including cancer, neurodegenerative diseases, and angiocardiopathy [85,98]. Researchers have been exploring the potential of targeting these pathways for therapeutic purposes [99].

Several UPS-targeted drugs have already entered the clinic, with proteasome inhibitors, such as bortezomib (Velcade), approved for multiple myeloma and mantle cell lymphoma [100,101,102]. This approval has sparked interest in developing more UPS inhibitors as effective cancer treatments. However, despite this clinical success, the efficacy of bortezomib in solid tumors is limited, and issues of drug resistance, relapse, and adverse side effects (e.g., peripheral neuropathy) substantially restrict its broader application [103,104,105]. This highlights the translational gap between hematologic and solid malignancies and the need for next-generation, more selective inhibitors.

Beyond proteasome inhibition, therapeutic interventions targeting microRNAs themselves have also been explored. For example, the synthetic miR-34a mimic MRX34 entered phase I clinical trials as the first miRNA-based therapeutic [5,106,107]. Although it demonstrated proof-of-concept, the trial was terminated due to immune-related adverse events, underscoring both the promise and the risks of directly manipulating miRNAs in patients. Together, these examples illustrate strategies that are validated at the preclinical or clinical level but still face major translational barriers.

By contrast, strategies aimed at targeting ZSWIM8, AGO proteins, or RNA-binding proteins (RBPs) to modulate TDMD remain largely conceptual, as these proteins have not yet been directly drugged and their systemic consequences are poorly understood. While ZSWIM8-directed TDMD inhibition may offer opportunities to stabilize tumor-suppressive miRNAs or to enhance antiviral immunity, these concepts require substantial mechanistic and translational validation. A major challenge is the identification of reliable diagnostic and prognostic biomarkers for miRNA dysregulation, alongside a deeper understanding of how miRNA–target interactions shape the biological characteristics of cancer [108,109].

Within this context, the UPS-dependent TDMD mechanism provides a novel framework to address these challenges, as the UPS directly regulates miRNAs through the TDMD pathway. Consequently, targeting components of the TDMD pathway within the UPS may offer several promising druggable nodes for therapeutic intervention [29,39]. Some of the key targets include ZSWIM8, AGO proteins, E3 ubiquitin ligases, proteasome, DUBs, chaperone proteins, and RBPs.

ZSWIM8 is a receptor of Cullin3 ligase and plays a crucial role in the recognition of AGO proteins within the TDMD pathway. It is involved in limiting the accumulation of certain miRNAs. Modulating the activity of ZSWIM8 could potentially regulate miRNA levels and restore their proper function in diseases. In addition to ZSWIM8, other members of the SWIM-type zinc finger family, including ZSWIM4, ZSWIM5, and ZSWIM6, have been implicated as potential cancer targets and biomarkers [108,109]. For example, ZSWIM4 shows preventive and diagnostic value for colorectal cancer and may be a therapeutic target for breast cancer [110]. ZSWIM8 is expressed in various cancer cells, including papillary thyroid carcinoma, non-small cell lung cancer, and human glioma, while ZSWIM5 is involved in embryonic and neural development [111,112,113]. Abnormal overexpression of ZSWIM6 protein has implications for lung cancer and prostate cancer [37,43,62,114].

AGO proteins are core components of the RNA-induced silencing complex (RISC), mediating miRNA loading, stability, and gene silencing functions. Within the TDMD pathway, AGO proteins serve as the direct substrates of ubiquitination by the ZSWIM8–CUL3 E3 ligase complex, which triggers their release of target miRNAs and subsequent degradation. Thus, selectively targeting AGO proteins in the context of TDMD could represent a strategy to modulate miRNA activity and to restore normal gene-regulatory function in disease conditions [39,115].

E3 ubiquitin ligases mediate substrate recognition and ubiquitin transfer, thereby determining the specificity of protein degradation. For instance, the ZSWIM8–CUL3 E3 ligase complex is responsible for ubiquitinating AGO proteins in the TDMD pathway, leading to miRNA turnover. Targeting disease-relevant E3 ligases may thus provide a means to regulate miRNA stability and expression levels [39].

The 26S proteasome is the major proteolytic machinery responsible for degrading ubiquitinated proteins. Modulating proteasome activity can indirectly affect miRNA stability by altering the degradation of miRNA-associated proteins. Although broad inhibition (e.g., by bortezomib) has shown clinical utility, selective modulation of proteasome subunits involved in TDMD may offer greater precision [116].

DUBs reverse ubiquitination and thereby stabilize target proteins. By removing ubiquitin from AGO proteins or other TDMD factors, DUBs may influence miRNA decay dynamics. Pharmacological targeting of specific DUBs could thus fine-tune miRNA turnover [117].

Chaperone proteins regulate protein folding and quality control, and several (e.g., HSP90) are known to stabilize AGO proteins and other RISC components. In the TDMD context, modulating chaperone activity could influence the stability of miRNA–protein complexes and thereby impact miRNA function [118].

RBPs interact with miRNAs at multiple stages, from biogenesis to decay, and play important roles in target recognition and turnover. Within TDMD, RBPs may act as cofactors that influence the accessibility or stability of AGO–miRNA complexes. Thus, targeting specific RBPs could provide another strategy to restore normal miRNA regulatory functions [119].

In summary, targeting the UPS and TDMD pathways holds great potential for cancer therapeutics, but further research is necessary. Overcoming challenges like drug resistance, side effects, limited efficacy, and the identification of biomarkers will be crucial for successful targeted therapy development. However, the potential drug targets within the TDMD pathway offer opportunities to restore miRNA homeostasis and function. By focusing on these key components, it may be possible to correct miRNA dysregulation and restore normal gene expression patterns in diseases associated with TDMD pathway dysfunction. Continued investigation and advancements in this field are needed to harness the full therapeutic potential of targeting the UPS and TDMD pathways.

## 6. Discussion and Outlook

The UPS-dependent TDMD mechanism plays a vital role in the accurate regulation and control of targeted therapeutic strategies. However, our understanding of the biological relevance of these interactions has been limited due to the lack of elaborate regulation and control models between the UPS and TDMD. Significant advances have been made in the models in recent years, enabling the study of the mechanism of miRNA degradation. It is now known that ZSWIM8, as a CRL substrate adaptor, recognizes and ubiquitinates AGO proteins to regulate miRNA abundance [29,39]. Understanding how AGO proteins modulate ZSWIM8 CRLs could provide new approaches to targeted therapeutic strategies for major diseases.

Since the discovery of miRNA, extensive research has focused on understanding their functional mechanism of miRNA. The regulation of miRNA abundance is a key factor that affects its functional mechanism. The turnover and biogenesis of miRNA together determine its abundance. However, many unanswered questions remain regarding the coordinated regulation of miRNA abundance between turnover and biogenesis. For example, it is not known if there are key coupling mechanisms mediating the balance between miRNA turnover and miRNA biogenesis.

The biogenesis of miRNA has spatiotemporal specificity, which determines the precise functional mechanism of miRNA in different physiological processes [21,120]. MicroRNAs are highly conserved, with more than one third of them found in nematodes and having homologs in vertebrate cells [121]. This provides a theoretical basis for studying the wide range of miRNA biogenesis and turnover mechanisms through model cells and/or animals [122]. MicroRNAs have complex regulatory networks in physiological processes and are widely involved in complex cellular signaling pathways [5,123]. Each miRNA may target several or even hundreds of potential genes. It has been observed that the regulated targets of a particular miRNA are not completely the same in different cells or different states of the same cell, indicating dynamic changes [124]. The factors that cause spatiotemporal specificity and dynamics of miRNA targets are still mysterious, with very little information available [62].

The TDMD pathway has been recognized as a specific and dynamic means of mRNA turnover. AGO is a core protein in the RISC complex, which plays an important role in the stability of miRNA abundance. Overexpression or knockdown of the AGO family proteins (AGO1-4) can lead to changes in miRNA abundance [125,126,127,128].

ZSWIM8-mediated TDMD has deep evolutionary roots. Deletion of the ZSWIM8 homolog in flies and worms stabilizes selective miRNAs, revealing numerous new uses for TDMD in vivo. Similarly, loss of function of the Cullin-Ring ligase ZSWIM8 accelerates the degradation of several miRNAs in mammalian, *Drosophila*, and nematode cells, thereby determining the half-life of most short-lived miRNAs. These and other results support a mechanistic model of TDMD in which the UPS-mediated hydrolysis of AGO-targeted proteins exposes miRNA degradation. These findings shed light on the mechanism of TDMD and extend its impetus to research on shaping miRNA levels in animals.

In vitro and in vivo, TDMD is usually accompanied by the addition of non-template uridine or adenosine to the 3’ end (tail) of miRNA and subsequent 3’ to 5’ exonucleolytic shortening (trimming). The coexistence of miRNA addition and trimming with TDMD, as well as many examples of 3’ uridylation controlling the stability of small RNAs, suggests that addition and trimming are central to TDMD. Studies by Han et al. (2020) and Shi et al. (2020) demonstrate that ZSWIM8-mediated TDMD and tail trimming pathways are separable: in ZSWIM8-deficient cells, miRNAs bound to TDMD triggers undergo extensive tailing and trimming, while synthetic Mir-7, protected from tailing and trimming by a 3’-end 2-O-methyl group, still triggers TDMD [29,39].

Currently, the TDMD mechanism has been identified as the primary mode of selective degradation, but some miRNAs are not sensitive to this mode, indicating the existence of other turnover mechanisms that need further study [29,39]. Recent research has not only provided new insights into miRNA biogenesis and turnover mechanisms but has also led to the discovery of exciting new mechanisms by which TDMD regulates miRNA turnover. However, many unanswered questions and unknown mechanisms remain. Further insights into the structure of miRNA-generated and turnover protein complexes are essential for a detailed understanding of these molecular mechanisms. New developments in cryo-electron microscopy will certainly accelerate these studies, allowing for the clarification of the common structure of RISC and ZSWIM8 Cullin-Ring, which may exist in different states or conformations and will be a major research direction for miRNA turnover.

Interactions between different cellular pathways and miRNA biogenesis and turnover have been reported, and it is likely that more of these types of connections will be unraveled. For example, the regulatory potential of RNA-binding proteins (RBPs) is closely related to miRNA biogenesis, and a wide range of miRNA regulatory layers can be predicted by RBPs. Additionally, the interaction between miRNA biogenesis and signal transduction through miRNA-processing enzyme phosphorylation is emerging as an important regulatory principle in healthy and cancerous tissues. Undoubtedly, in the near future, many exciting new discoveries will contribute to understanding the role of miRNA biogenesis in diseases such as cancer.

## Data Availability

The original contributions presented in this study are included in the article. Further inquiries can be directed to the corresponding authors.
